# Budding of Enveloped Viruses: Interferon-Induced ISG15—Antivirus Mechanisms Targeting the Release Process

**DOI:** 10.1155/2012/532723

**Published:** 2012-05-17

**Authors:** Eun Joo Seo, Jonathan Leis

**Affiliations:** Department of Microbiology and Immunology, Northwestern University Feinberg School of Medicine, Ward 4-065, 303 E. Chicago Avenue, Chicago, IL 60611, USA

## Abstract

Pathogenic strains of viruses that infect humans are encapsulated in membranes derived from the host cell in which they infect. After replication, these viruses are released by a budding process that requires cell/viral membrane scission. As such, this represents a natural target for innate immunity mechanisms to interdict enveloped virus spread and recent advances in this field will be the subject of this paper.

## 1. Budding of Retroviruses

**Figure 1:**
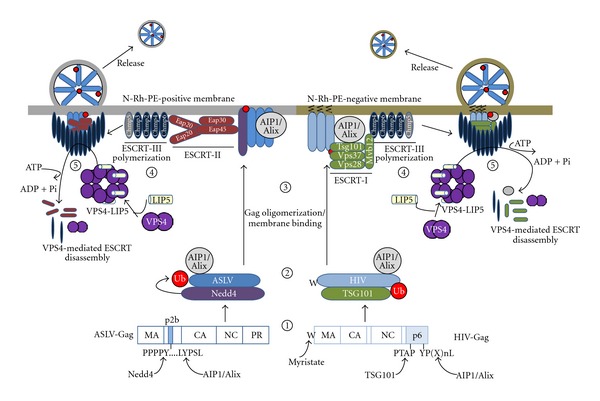
Parallel pathways used by ASLV and HIV-1 Gag to bud from cells. Retroviruses recruit components of the ESCRT machinery to assemble budding complexes. Step 1: HIV-1 and ASLV Gag L-domains bind to Tsg101 and Nedd4, respectively. They also bind the Alix adaptor protein. Whether these initial interactions take place in the cytosol or at the plasma membrane remains to be defined. Step 2: Nedd4 mediates ubiquitination of ASV Gag. HIV-1 Gag is ubiquitinated by an unidentified E3 ligase. Step 3: Gag oligomerization in the cytosol increases membrane avidity and in conjunction with the M domain signal at the N-terminus of Gag targets the polyproteins to sites of assembly/budding on the plasma membrane. ASLV Gag assembles on rhodamine labeled phosphatidylethanolamine (N-Rh-PE)-positive, endosome-derived membranes. HIV-1 Gag assembles on N-Rh-PE-negative membranes. Step 4: The ASLV Gag/Nedd4/Alix complex recruits ESCRT-II proteins while the HIV-1 Gag/Tsg101/Alix complex recruits the remainder of the ESCRT-I proteins. Each early budding complex then recruits the same ESCRT-III machinery which promotes the membrane scission to release VLPs from the cellular membranes. Step 5: The ESCRT-III subunits recruit the AAA ATPase, VPS4, and the coactivator protein LIP5 to mediate the disassembly of membrane-bound ESCRT complexes concomitant with the budding process.

Retroviruses, such as human immunodeficiency virus type 1 (HIV-1) and avian sarcoma/leukosis virus (ASLV), bud from cells using a similar mechanism (see Figure 1). Monoubiquitination of viral Gag polyproteins, catalyzed by an E1, E2, and E3 ubiquitin ligase complex, is important in the process where the ubiquitin most likely serves as a ligand for assembly of various protein budding complexes [1–5]. A recent study, however, has presented evidence that either ubiquitination of Gag or ubiquitination of transacting proteins can be used to assemble downstream virus-budding complexes [6]. Ubiquitin is a 76-amino-acid cell regulatory protein that is conjugated to proteins at lysine residues. Free ubiquitin in the cytosol is activated in an ATP-dependent reaction by an E1 enzyme, which then transfers the ubiquitin to a cysteine residue of an E2 ubiquitin-conjugating enzyme. The E2 protein interacts with a specific E3 ubiquitin ligase, which selects the target protein for the covalent transfer of the ubiquitin.

While the mechanisms of release of HIV-1 and ASLV are similar, there is evidence that these viruses traffic through different cell membranes in the process. This is based on the facts that ASLV passes through membranes containing rhodamine labeled phosphatidylethanolamine, which gets incorporated into released virus-like particles, while HIV-1 does not [7], the two viruses utilize different early ESCRT complexes as outlined hereinafter, and HIV-1 and ASLV Gag can colocalize with cholesterol-rich membranes, referred to as rafts. Nonetheless, when cholesterol is depleted from cells by treatment with methyl-*β*-cyclodextrin, release of HIV-1 Gag [8] but not ASLV Gag (Zhang and Leis, unpublished) virus-like particles is blocked.

 Viral polyproteins enter the different budding pathways by recruiting specific host cell proteins, Nedd4, an E3 ubiquitin ligase, for ASLV [9], Tsg101, an inactive homologue of the E2 ubiquitin-conjugating enzyme, for HIV-1 [10], and AIP1 or Alix for both [11, 12]. These proteins bind to small proline-rich sequences, referred to as L-domain. Nedd4 binds to the “PPPY” core motif in the p2B region of ASLV Gag and Tsg101 binds to the “PTAP” core motif in the p6 region of HIV-1 Gag [13–15]. AIP1 binds to a “LYP (X_*n*_)L” core motif in the same regions of p2B and p6, respectively, and where X is any amino acid and *n* is a number of residues [11, 12]. EIAV uses the LYPXL sequence in the p9 region of Gag as a high affinity site to bind AIP1/ALIX [16]. The PPPY and PTAP L-domains are functionally exchangeable among viruses [7, 17, 18]. In the case of HIV-1, if the L-domain sequences in p6 are mutated, budding of the virus can be rescued by overexpression of a Nedd4-like ubiquitin ligase (Nedd4L or 2s) indicating that an alternative exit process was used, though the mechanism of rescue is not understood because of a lack of the PPPY motif in HIV-1 Gag [19–21]. Overexpression of AIP1/ALIX can also rescue the effects of PTAP mutations [20]. In some cases, viruses such as Moloney murine leukemia virus, Mason Pfizer monkey virus, and HTLV-I have both PTAP and PPPY motifs in Gag suggesting that they may use both egress pathways [22, 23]. However, when two L-domains are present, one appears to be dominant [22].

 Tsg101 is known to be involved in membrane vesicle biogenesis and cytokinesis [24, 25]. It forms part of the cellular endosomal sorting complex required for transport (ESCRT), which contains at least 20 proteins that assemble into several complexes referred to as ESCRT-0, -I, -II, and -III [26]. HIV-1 specifically uses ESCRT-I but not ESCRT-II while ASLV utilizes ESCRT-II but not ESCRT-I proteins to assemble early budding complexes [27–29]. AIP1 is an adaptor protein for both. The early budding complexes then recruit the same ESCRT-III protein complex, which is responsible for the mechanical deformation that causes membrane scission to release particles [30]. The ESCRT-III complex contains 11 different charged multivesicular body proteins (CHMPs) [31]. Several of these contain MIT-interaction motifs (MIMs), which are the binding site for the vacuolar protein sorting protein 4 (VPS4) through interaction with a microtubule-interacting and transport domain (MIT) [32, 33]. There are two types of MIM domains known; MIM1 is found in CHMP1A, 1B, 2A, and 2B and MIM2 is found in CHMP4A-C and CHMP6 [34, 35]. VPS4 is normally localized in the cytosol as a homodimer. When recruited to the membrane, it forms a double hexamer ring structure in the presence of a coactivator protein, called LIP5 [32, 36]. This conformational change activates its AAA ATPase. When ATP is hydrolyzed, VPS4 and the ESCRT-III complex are disassembled into the cytosol, promoting complex turnover which increases the efficiency of the budding process. A point mutation, E228Q, which inactivates the ATPase, produces a VPS4 protein that acts as a dominant negative inhibitor to virus release [7, 23, 27]. LIP5 is delivered to membranes and VPS4 by binding to several ESCRT-III proteins including CHMP1B, 2A, 3, and 5 [37]. CHMP5 differs from the other ESCRT-III proteins in that its LIP5 binding site is unique and it does not directly interact with VPS4 [38].

## 2. Other Viruses Use an ESCRT-Dependent Budding Process

 Many other enveloped viruses have the same requirements for membrane scission to release particles and employ similar mechanisms to those used by retroviruses to drive the release process. These include arena- [39, 40], filo- [2, 41, 48–51], flavi- [52, 53], hepadna- [42], herpes- [43, 54], rhabdoviruses [46, 47, 55], and some paramyxoviruses [44, 45, 56]. Each virus contains L-domain sequences in their structural proteins and the release process is dependent upon the ESCRT-III/VPS4 ATPase complex. This was demonstrated experimentally by either directly mutating L-domain sequences within the viral proteins or by dominant negative interference caused by exogenous expression of the VPS4 (E228Q) mutant. While many of the L-domain sequences have been identified (see Table 1), some have not. For example, even though budding of the flavivirus HCV is dependent upon the ESCRT-III/VPS4 complex, the L-domain sequences involved are not known [52, 53]. Among herpesvirus proteins, there are multiple candidates for possible L-domain sequences. Which is used is not clear. However, there is evidence that the budding process is independent of Tsg101 suggesting that this virus uses a PPPY-dependent pathway [54]. L-domains different from those used by retroviruses include the FPIV core sequence in the paramyxoviruses SV5 and mumps (MuV) [44, 45], though the mechanism of the release is not understand.

## 3. ESCRT-Independent Release

 While paramyxoviruses such as mumps and SV5 use an ESCRT-dependent pathway for release [44, 45], there are reports that measles and respiratory syncytial virus use an as yet unknown ESCRT-independent pathway for budding [57, 58]. There is also a report that human cytomegalovirus uses an ESCRT-independent release mechanism [59], based solely on siRNA suppression data. However, a subsequent report demonstrated that release of CMV was blocked by a dominant negative mutant of VPS4 but not wild type VPS4 arguing that the virus does use the ESCRT-dependent mechanism [60]. Similarly, there is a report that rhabdoviruses use a VPS4-independent release pathway [61] and a subsequent publication demonstrating that budding was also VPS4 dependent [55].

 Some strains of influenza virus have evolved a completely different mechanism for cell release that employs the virus matrix protein (M), haemagglutinin, and neuraminidase (the latter being two major glycoproteins of the virus) [62–66]. Virus budding is initiated by haemagglutinin and neuraminidase association with the viral M1 protein at lipid rafts inside of the cell. M1 then recruits the M2 ion channel protein to catalyze the scission from the cell membrane leading to budding. A mutation introduced into an amphipathic helix of M2 results in a protein incapable of membrane scission blocking virus release. The neuraminidase facilitates the release of VLPs by cleaving the terminal sialic acid from cell membrane glycan [63, 66].

## 4. Interferon Regulation of the ESCRT-Dependent Virus Budding

 To control virus infections, host cells have evolved many different antiviral immune mechanisms as well as mechanisms that target viral replication directly [67, 68]. Some of the latter are based on synthesis of enzymes or restriction factors, such as APOBEC3, TRIM5, and tetherin that interfere with virus replication functions [69, 70]. APOBEC3 is a cytosine deaminase that catalyzes the conversion of cytosine to uricil and ammonia [71]. TRIM5 is a ring finger E3 ubiquitin ligase protein that mediates an early block to retrovirus infection by interfering with virus uncoating [72, 73]. Tetherin (CD317 or BST2) is an interferon-induced protein that blocks release of envelope viruses including Lassa and Marburg [74] and HIV-1 [75, 76] by preventing diffusion of virus particles after budding. It also blocks cell-to-cell transfer of virus [77]. While the mechanism of how tetherin works is not fully understood, its association with membranes from which viruses bud and its propensity to dimerize suggests that it might act as a nonspecific protein-based tether to fix particles to the membranes.

Many of the cellular antiviral mechanisms are initiated by induction of interferon (IFN). There are three groups of IFNs (types I, II, and III), classified by the IFN receptor complex to which they bind. It is the type I IFNs which primarily regulate the antiviral response. For instance, recombinant forms of type I IFNs demonstrate therapeutic potential for the treatment of chronic hepatitis B and C infections [78]. Some of the known interferon-induced genes include PKR/2′5′ oligoadenylate synthetase, interferon-stimulated gene 15 (ISG15), tetherin, and MxA [79].

ISG15 is one of the more abundant proteins induced by interferon expression which is associated with antiviral effects [80–88]. It has a molecular mass of about 15 kDa and is a covalent dimer homologue of ubiquitin [89, 90]. It can be conjugated to several hundred different proteins in cells [91–93] catalyzed by an interferon-induced ISG15 E1 (Ube1L) [94, 95], E2 (UbcH8) [96, 97], and E3 (Herc5) [98, 99] ligase complex. The targets are largely abundant constitutively expressed proteins involved in diverse cellular functions, with the exception of around 15 proteins which are themselves interferon induced [92, 99, 100]. A common sequence that serves as an ISG15 covalent attachment site has not been identified. For recent reviews of ISG15, see [87, 101, 102]. Retrovirus release from cells is blocked by interferon treatment or exogenous expression of ISG15 in cells [49, 83, 84, 103, 104]. Also, ISG15 is conjugated to the influenza A virus NS1A protein to block its replication [93]. ISG15 knockout (ISG15^−/−^) mice show increased susceptibility to influenza, herpes, chikungunya (alphavirus), and sindbis virus infections as measured by decreased survival rates compared to wild type mice [105, 106]. In addition, expression of a conjugation-deficient form of ISG15 fails to protect ISG15^−/−^ mice from sindbis infection [94, 105, 107].

## 5. Mechanism of Inhibition of Enveloped Virus Budding by ISG15 Expression in Cells

### 5.1. Blockage to Early Virus-Budding Events

When ISG15 is expressed in cells, there is a block early in the virus-budding process that appears to be virus specific. For example, for HIV-1 infections, ISG15 expression prevents the L-domain in the p6 region of HIV-1 Gag from recruiting Tsg101 [103]. While Tsg101 and Gag are not ISGylated, the E2 ubiquitin-conjugating enzyme is [103]. In contrast, for Ebola and other enveloped viruses, ISG15 expression inhibits the Nedd4 E3 ubiquitin ligase by binding to the E3 subunit thereby averting its interaction with the E2 ubiquitin-conjugating enzyme. This prevents ubiquitination of viral proteins required for the release process [49, 92, 108]. Mice lacking Ube1L are as susceptible to Chickungunya virus infections as wild type mice indicating that ISG15 conjugation to protein is not involved [106].

### 5.2. Blockage to Late Virus-Budding Events

**Figure 2:**
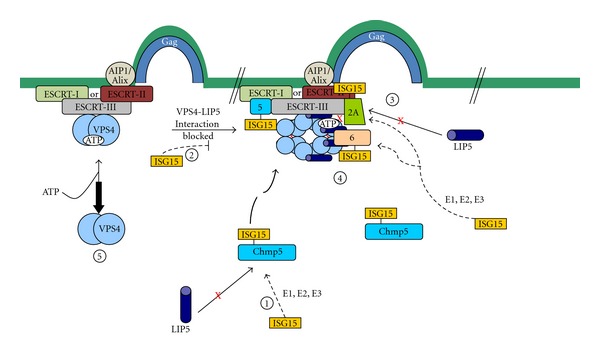
The mechanism of inhibition of budding late in the process mediated by ISG15 expression in cells. Step 1: The ESCRT-III protein, CHMP5, binds to LIP5 in the cytosol. In the presence of ISG15-specific E1, E2, and E3 ISG15 ligase complex, ISG15 is conjugated to CHMP5 and the binding to LIP5 is lost. Step 2: The CHMP5-ISG15 conjugate accumulates in the budding complex on the membrane. The interaction between VPS4 and its coactivator LIP5 is blocked preventing activation of the ATPase through formation of the VPS4/LIP5 double hexamer structure. Step 3: CHMP2A is ISGylated in the presence of ISG15-CHMP5. This results in the loss of its binding to LIP5 and weakens its direct interaction to VPS4. Step 4: CHMP6 is ISGylated in the presence of ISG15-CHMP5. This results in weakening of its binding to VPS4. Step 5: VPS4 is released into the cytosol and virus budding is arrested. (This figure was modified from Figure 7 in [83].)

Disruption of the late release process represents a more general antiviral mechanism which requires conjugation of ISG15 to several ESCRT-III proteins (see Figure 2). The initial target for ISGylation is the ESCRT-III-associated LIP5 binding protein, CHMP5 [84]. This protein is not ISGylated when a GG to AA mutant of ISG15, which cannot be conjugated to protein, is expressed in cells [83]. When CHMP5 is ISGylated, it no longer binds LIP5 and VPS4 is released from and/or not recruited to the budding complex in the membrane resulting in a budding defect [84]. CHMP5 like VPS4 is a cytoplasmic protein, which is recruited to the viral budding complex by interaction with several ESCRT-III proteins [34, 91]. When CHMP5 becomes ISGylated, it accumulates in the membrane fraction [83, 84]. ISG15 is also conjugated to CHMP2A, another ESCRT-III protein that binds LIP5. Like CHMP5, ISGylation of CHMP2A prevents its binding to LIP5. In contrast, the binding of LIP5 to CHMP3 was not affected under these same conditions. Because ISGylation of CHMP5 and CHMP2A correlates with the release of VPS4 from the budding complex, it suggests that CHMP5 and CHMP2A but not CHMP3 are the primary physiological delivery systems of LIP5 to VPS4.

 In addition to compromising its ability to bind LIP5, CHMP2A-ISG15 shows significant decreases in binding to VPS4. In contrast, changes in CHMP1B-VPS4 and CHMP3-VPS4 interactions were not detected [83, 84]. ISG15 is also conjugated to two other ESCRT-III proteins, which do not bind LIP5, CHMP4B, and CHMP6 [83]. Both of these proteins bind to VPS4 [34], and when ISGylated, their binding to this protein is weakened [83]. Thus the release of VPS4 into the cytosol is caused by the sum of ISGylation of several ESCRT-III proteins along with the lack of available LIP5 to form the stable VPS4/LIP5 double-hexamer-ring structure in the membrane. ISGylation of CHMP5 is pivotal to initiate the general antiviral mechanism because removal of CHMP5 from cells using specific but not nonspecific siRNAs prevents ISG15 from inhibiting virus release. In addition, ISGylation of the other ESCRT-III proteins including CHMP2A and CHMP6 is dependent upon prior ISGylation of CHMP5 [83].

 ISG15 can be induced in cell culture by treatment with interferon or by introduction of the ISG15 expression system. For example, release of ASLV from DF-1 cells was blocked to the same extent by either treatment [83]. If CHMP5 expression was significantly lowered in these cells by using specific targeting siRNAs, the inhibition of virus release caused by either treatment was only partially restored. This is not surprising because ISG15 expression acts to inhibit both early steps not dependent upon CHMP5 as well as late steps dependent upon CHMP5. Based upon the degree of rescue, it was estimated that about half of the inhibition to release of ASLV was caused by blocking CHMP5-indpendent early steps and half by blocking the late CHMP-dependent steps in the budding process [83]. The finding that many different ESCRT-III proteins are ISGylated and that this results in changes in the binding properties of each protein to LIP5 or VPS4 strongly suggests that interferon-induced expression of ISG15 will shut down normal cellular endocytosis and MVB biogenesis.

## 6. Virus Defense Mechanisms

 Ubiquitin metabolism is very important both to the spread of enveloped virus infections and for bypassing host restrictions. As mentioned previously, monoubiquitination of viral structural proteins or transacting proteins is important for recruitment of the ESCRT complexes responsible for membrane scission leading to budding. In contrast, polyubiquitination is used by viruses to remove host restriction factors. For HIV-1, the action of tetherin is downregulated by expression of the Vpu protein, which targets this protein for degradation via a *β*-TrCP2-dependent pathway [109]. On the other hand, release of the human herpesvirus Kaposi's sarcoma-associated herpesvirus (HHV8) is also blocked by tetherin expression. In this case, it is counteracted by expression of an E3 ubiquitin ligase, K5, which catalyzes the polyubiquitination of tetherin leading to its degradation [110]. When APOBEC3 interacts with the HIV-1 viral infectivity factor (Vif), it also becomes polyubiquitinated and is degraded by the ubiqutin-dependent proteasome system [71].

 Several enveloped viruses encode proteins that target the ISGylation pathway directly in order to propagate [82]. For example, the NS1 protein of influenza B virus prevents the interaction of ISG15 with Ube1L to inhibit ISGylation [95, 111, 112]. A crystal structure of human ISG15 in complex with an N-terminal fragment of NS1B has been solved [113]. One can speculate that these results suggest that some strains of the flu virus either now or in the past utilized an ESCRT-dependent pathway for release. Other examples include nairoviruses and arteriviruses, which encode ovarian tumor domain-containing deubiquitinating enzymes that remove ISG15 from substrates [81, 114, 115]. A vaccinia virus E3 protein also prevents the antiviral action of ISG15 [116].

## 7. Clinical Perspectives

 The mechanism for blocking release of retroviruses from cell membranes has broad implications for many enveloped virus infections. If drugs can be identified that prevent viruses from recruiting ESCRT proteins to budding complexes, we would have compounds that would act to slow down the spread of many pathogenic enveloped viruses giving the immune system time to clear the infection. Such drugs would complement natural immune mechanisms that target virus budding. This could be a clinical game changer world-wide for treatment of viral infections and associated diseases in humans.

## Figures and Tables

**Table 1:** L-domains found in enveloped virus proteins, which bud from the cell membrane in an ESCRT-dependent process: Lassa fever virus (LFV) [39], lymphocytic choriomeningitis virus (LCMV) [39, 40], Ebola virus (EboV) [2], Marberg virus (MarV) [41], hepititus B virus (HBV) [42], Herpes simplex virus, type 1 (HSV-1) [43], Simian virus, type 5 (SV5) [44], Mumps virus (MuV) [45], avian sarcoma leukemia virus (ASLV) [11, 14, 15], human immunodeficiency virus, type 1 (HIV-1) [2, 10, 27], human T-lymphotropic virus, type 1 (HTLV-1) [2], equine infectious anemia virus (EIAV) [12, 16], vesicular stomatitis virus (VSV) [46], and rabies virus (RV) [47].

Virus species	Virus	Protein	Amino acid sequence containing L-domain
Arenavirus	LFV	Z	AA**PTAP**PTGAADSI**PPPY**SP
	LCMV	Z	TAPSS**PPPY**EE

Filovirus	EboV	VP40	MRRVIL**PTAPPEY**MEAI
	MarV	VP40	NTYMQYLN**PPPY**ADHS

Hepadnavirus	HBV	Core protein	**PPAY**

Herpesvirus	HSV-1	E	**PPTY**

Paramyxovirus	SV5	M	QSIKA**FP**I**V**INSDG
	MuV	M	RLNA **FPIV**MGQ

Retrovirus	ASLV	p2B of Gag	ATASAP**PPPY**VGSG**LYP**S**L**
	HIV-1	p6 of Gag	PE**PTAPP**FLQSRPE**PTAP**PEES..E**LYP**LTS**L**R
	HTLV-I	MA of Gag	DPQI**PPPY**VE**PTAP**
	EIAV	p9 of Gag	QNL**YPDL**SEIK

Rhabdovirus	VSV	M	LGIA**PPPY**EEDTSMEYA**PSAP**
	RV	M	DDLWL**PPPEY**VPLKEL
